# Speedy stomata of a C_4_ plant correlate with enhanced K^+^ channel gating

**DOI:** 10.1111/pce.14775

**Published:** 2023-11-27

**Authors:** Fernanda A. L. Silva‐Alvim, Jonas Chaves Alvim, Andy Harvey, Michael R. Blatt

**Affiliations:** ^1^ Laboratory of Plant Physiology and Biophysics, Bower Building University of Glasgow Glasgow UK; ^2^ Physics & Astronomy University of Glasgow Glasgow UK

**Keywords:** electrophysiology, *Gynandropsis gynandra*, light, photosynthetic, stomatal kinetics, water use efficiency

## Abstract

Stomata are microscopic pores at the surface of plant leaves that facilitate gaseous diffusion to support photosynthesis. The guard cells around each stoma regulate the pore aperture. Plants that carry out C_4_ photosynthesis are usually more resilient than C_3_ plants to stress, and their stomata operate over a lower dynamic range of CO_2_ within the leaf. What makes guard cells of C_4_ plants more responsive than those of C_3_ plants? We used gas exchange and electrophysiology, comparing stomatal kinetics of the C_4_ plant *Gynandropsis gynandra* and the phylogenetically related C_3_ plant *Arabidopsis thaliana*. We found, with varying CO_2_ and light, that *Gynandropsis* showed faster changes in stomata conductance and greater water use efficiency when compared with *Arabidopsis*. Electrophysiological analysis of the dominant K^+^ channels showed that the outward‐rectifying channels, responsible for K^+^ loss during stomatal closing, were characterised by a greater maximum conductance and substantial negative shift in the voltage dependence of gating, indicating a reduced inhibition by extracellular K^+^ and enhanced capacity for K^+^ flux. These differences correlated with the accelerated stomata kinetics of *Gynandropsis*, suggesting that subtle changes in the biophysical properties of a key transporter may prove a target for future efforts to engineer C_4_ stomatal kinetics.

## Summary Statement

Compared with Arabidopsis, the outward‐rectifying K^+^ channels in guard cells of the C_4_ plant *Gynandropsis gynandra* incorporate gating that is displaced to favour enhanced K^+^ efflux and promote K^+^ influx. These characteristics are likely to underpin the faster stomatal that are observed in the C_4_ plant.

## 1 INTRODUCTION

In C_3_ plants the photosynthetic efficiency (ratio of carboxylation to oxygenation) decreases rapidly at high temperatures (Laing et al., 1974) and periods of drought because the stomatal closure limits the CO_2_ uptake (Simpson et al., 2022). C_4_ metabolism is one of the CO_2_‐concentrating mechanisms that has evolved to increase the concentration of CO_2_ at the active site of Rubisco (Hatch, 1971; Hatch & Slack, 1966). C_4_ plants are usually more resilient than C_3_ plants to stress, exhibiting smaller stomatal conductances, a higher water use efficiency (WUE) and higher photosynthesis rates when compared with C_3_ plants (Osborne & Sack, 2012; Pardo & VanBuren, 2021; Yadav & Mishra, 2020).

Stomata pores on the leaf surface control gas exchange with the external environment and react by opening and closing in response to environmental conditions, notably light, CO_2_ and humidity. Stomata must promote CO_2_ diffusion into the leaf for photosynthetic carbon assimilation while controlling water loss via transpiration (Lawson & Blatt, 2014). Stomatal conductance (*g*_s_) has been inversely correlated to the size of the stomata and directly correlated to stomatal density (Bertolino et al., 2019; Franks & Beerling, 2009; Harrison et al., 2020; Wei et al., 2020; Xiong et al., 2022). When comparing C_3_ and C_4_ species, however, there is little consensus. For example, Taylor et al. (2012) observed among C_4_ species a lower stomatal density compared with related C_3_ species, but there was no significant difference in the size of the stomata. By contrast, a significant difference in density, size and stomatal length was reported between the C_4_ and C_3_ species within the genus *Flaveria* (Zhao et al., 2022), while among C_3_ and C_4_ grasses no differences were reported in stomatal size or density (Israel et al., 2022).

Fewer studies have compared the dynamic responses of stomata between related C_3_ and C_4_ species. Stomata of C_4_ plants were reported to open and close more rapidly than C_3_ species in response to light (Israel et al., 2022; McAusland et al., 2016; Ozeki et al., 2022) and similar characteristics were observed in C_4_ species of *Flaveria* in response to CO_2_ (Huxman & Monson, 2003). Such behaviour could facilitate carbon assimilation and enhance WUE (McAusland et al., 2016). However, other studies have suggested that C_4_ species perform poorly when light varies (Li et al., 2021; Slattery & Ort, 2019).

Stomatal movements are regulated by the osmotic solutes that generate turgor pressure in the pair of guard cells surrounding the stomatal pore (Jezek & Blatt, 2017; Lawson & Blatt, 2014; Vialet‐Chabrand & Lawson, 2019; Willmer & Fricker, 1995). The transport of solutes, especially of K^+^, facilitates changes in guard cell turgor that drive these dynamics (Ankit et al., 2022; Jezek & Blatt, 2017; Kumar et al., 2020). For stomatal opening, plasma membrane H^+^‐ATPases mediate H^+^ efflux from the cytosol to hyperpolarize the membrane potential, negative inside, and activate inward‐rectifying K^+^ channels (K_in_) to promote K^+^ uptake. Potassium accumulation is balanced by the production of malate^2−^ and the uptake of Cl^−^ and NO$\begin{array}{c} -\\ 3 \end{array}$ that, together, drive osmotic water influx to increase stomatal turgor (Jezek & Blatt, 2017; Lawson & Blatt, 2014; Willmer & Fricker, 1995). During stomatal closure, inhibition of plasma membrane H^+^‐ATPases and activation of anion channels promote membrane depolarisation, efflux of K^+^ through outward‐rectifying K^+^ channels (K_out_), and of Cl^−^, NO$\begin{array}{c} -\\ 3 \end{array}$ and malate^2−^ through anion channels, with the loss of water and turgor of the guard cells (Jezek & Blatt, 2017; Lawson & Blatt, 2014; Roux & Leonhardt, 2018; Willmer & Fricker, 1995).

Understanding how ion channel gating translates to stomata mechanics could help guide improvements in crop resilience and yield. Much knowledge is available pertaining to channel gating, its kinetics and their implications (Blatt, 1992; Blatt & Armstrong, 1993; Brearley et al., 1997; Jezek & Blatt, 2017; Marten et al., 2008; Roelfsema et al., 2001; Thiel et al., 1992; Wang et al., 2017). However, to date, the vast majority of work has focused on these characteristics in C_3_ species. With few exceptions (Bauer et al., 2000; Fairley‐Grenot & Assmann, 1993; Gao et al., 2017; Natura & Dahse, 1998; Philippar et al., 1999, 2003; Terry et al., 1992), our knowledge of transport and its integration in C_4_ species is fragmentary at best. Yet, there is some evidence that points to differences in ion transport between C_3_ and C_4_ species (Gao et al., 2019; Israel et al., 2022; Su et al., 2005).

*Gynandropsis gynandra* was proposed as a C_4_ model plant (Brown et al., 2005; Marshall et al., 2007) in addition to *Zea mays* and *Flaveria* species (Ermakova et al., 2020; Sales et al., 2021; Simpson et al., 2022), and presents several advantages. Among these, *G. gynandra* is phylogenetically the closest C_4_ species to the model C_3_ plant *Arabidopsis thaliana* (Hoang et al., 2023). Cleomaceae and Brassicaceae are sister clades that share a common ancestral gene duplication. The clades have retained a high level of genome synteny and collinearity, although each clade underwent one further genome duplication event, thus separating Arabidopsis from Brassica species on the one hand and *G. gynandra* from another Cleomaceae species, *Tarenaya hassleriana* on the other hand (Cheng et al., 2013; Hoang et al., 2023; Schranz & Mitchell‐Olds, 2006). Additionally, *G. gynandra* harbours a small genome (Hoang et al., 2023), and it has a relative short life cycle with a high seed yield (Brown et al., 2005). Here we have asked whether there are outstanding differences in the gating of the predominant K^+^ channels that affect stomata dynamics and could help explain the WUE of this C_4_ plant by comparison with Arabidopsis.

## 2 MATERIALS AND METHODS

### 2.1 Plant growth

Plants were grown in a growth chamber under controlled environment (Sanyo FitoTron), either under short‐day or long‐day conditions. Short‐day had 9 h of light at 22°C and 60% relative humidity (RH), and 15 h of dark at 18°C and 70% RH. While long‐day conditions had 16 h of light at 26°C and 60%, and 8 h of dark, 22°C and 70% RH.

*Arabidopsis thaliana* Columbia 0 wild‐type seeds were sown in 10 cm pots containing the nutrient‐rich Levington F2 + S3 compost (Coulders). After sowing on soil, seeds were stratified at 4°C, for 48 h in the dark and left to germinate under a plastic lid (>95% RH) for 2 weeks. Seedlings were individually transplanted into 6 cm pots with the same compost and covered with polyester mesh (mesh width 0.3 mm). Pots were kept under propagator with mesh fabric (mesh of 200 μm diameter) over the sides of the covers to permit free air exchange. Plants were grown under 120 μmol m^−2^ s^−1^ of light in short‐day conditions.

Seeds of *G. gynandra* were provided by Prof. Julian M Hibberd (Department of Plant Sciences, Downing Street, University of Cambridge, Cambridge CB2 3EA, UK). *G. gynandra* seeds were germinated on moist soil and dark for 72 h. After germination, plants were cultivated in 10 cm pots with Levington Advance Pot & Bedding M3 Compost (ICL Specialty Fertilizers) supplemented with vermiculite (Gro‐sure, Westland Horticulture), at a rate of 4:1. Plants were grown under 160 μmol m^−2^ s^−1^ white light and long‐day conditions. Leaves from 4‐ to 6‐week‐old plants were used in the analyses.

### 2.2 Stomatal assays

Stomata size and density were recorded from fresh abaxial epidermal peels submerged in water. Stomata were imaged using AmScope Microscope Digital Camera (MU1803 USB3.0 18MP Colour) attached to a Zeiss Axiovert 35 Inverted Microscope with a 10× or 20× Objective. Measurements of length and width were carried out individual stoma, and results are reported as means ± SE of *n* > 50 stomata from at least four different plants. For the stomata density analyses, stomata were counted on at least two areas of 0.20 mm^2^. Sizes were tracked for individual stomata and quantified using ImageJ version 2.30 (image.nih.gov/ij/).

### 2.3 Gas exchange

Gas exchange measurements (CO_2_ and H_2_O flux) were carried out using Li‐COR 6800 gas exchange systems (Lincoln) equipped with a Multiphase Flash™ Fluorometer (6800‐01 A, LI‐COR). Measurements were performed on individual leaves attached to the plants after at least 60 min of acclimation in the leaf cuvette. A balance of 10% blue and 90% red illumination was set to provide the necessary photosynthetic photon flux density, using the light‐emitting diode array of the top‐side cuvette. Leaf chamber temperature was controlled at 22.5°C and air RH at 60%. The flow was set at 200 µmol s^−1^ (pump auto), 0.2 kPa and boundary layer conductance of 2 mol m^−2^ s^−1^. The atmospheric CO_2_ mixing ratio in the cuvette was controlled to be 400 µbar or as stated in the figure legends. Gas‐exchange parameters were calculated according to von Caemmerer and Farquhar (1981). At least four plants per species or genotype were measured on different days at the same point of the diurnal cycle. Data were normalised for leaf area using ImageJ v.1.51 (rsbweb.nih.gov/ij/).

Experiments were setup to use either CO_2_ or light as a parameter to induce stomatal movement and the transpiration rates were continuously recorded. Data were logged every 1 min for all plants.

To quantify differences in stomatal kinetics, we fitted stomata conductance (*g*_s_) relaxations on opening (Equation 1) and closing (Equation 2) to first‐order exponential functions with an offset (exponential decay, single, 3 parameters):

(1) $$f\left(x\right)=y0+a\times \left(1-{\text{exp}}^{\left(-b\times x\right)}\right),$$

(2) $$f\left(x\right)=y0+a\times {\text{exp}}^{\left(-b\times x\right)},$$

where *y*0 is the *y* value when *x* (time) is zero and is expressed in the same units as *y*, a is the difference between *y*0 and the final steady state at infinite times expressed in the same units as *y*, and *b* is the rate constant expressed in the reciprocal of the *x* axis units. If *x* is in minutes, then b is expressed in inverse minutes (min^−1^).

The *t*_1/2_ is the time, in minutes, taken by the stomata to achieve 50% change from the starting point to the final steady‐state g_s_, after changing external light or CO_2_ partial pressure. The halftime for stomatal opening and closure were calculated from the constants (*b*) according to:

(3) $$t_{1/2}=\;\ln\;2/b.$$

### 2.4 Guard cell electrophysiology

Microelectrodes were pulled using borosilicate glass capillary (Dial Glassworks) using a modified two stage PD5 horizontal puller (Narashige Scientific Instrument Lab). The capillaries, typically between 3 and 5 cm in length, were clamped in the puller, heated for 15 s and twisted through 360°. The barrels were cooled for 40 s, heated a second time and pulled out through a normal pull cycle. Heater and magnet settings were adjusted to optimise shape and tip size, which were controlled by the time of pulling. To be able to insert the microelectrodes into the half‐cells, the back ends of the barrels were heated in small gas flame and bended in a S‐shape using forceps. Microelectrodes were kept in a sealed glass container to avoid breakage and contamination of the fine tips (Blatt, 1987; Wang, 2013). Microelectrodes were filled with 200 mM K^+^‐acetate (pH 7.4) to minimise interference arising from anion leakage from the microelectrode and coated with paraffin to reduce electrode capacitance (Blatt, 1987). For *A. thaliana* guard cells, the filling solution was equilibrated with Ca^2+^ buffer 1,2‐bis(2‐aminophenoxy)ethane‐N,N,N′,N′‐tetraacetic acid, to prevent leakage of Ca^2+^ from the microelectrode. For *G. gynandra* guard cells microelectrodes were pulled to give smaller resistances than previously used with *A. thaliana* (Wang et al., 2012).

Half‐cells filled with 1 M KCl were used to connect the microelectrodes to the amplifiers, and were home‐made as previously described (Blatt, 1987; Wang, 2013). Electrical contact between the reference half‐cell and cell bath solution was made by a salt bridge comprising polythene tubing filled with 2% agar dissolved in 1 M KCl.

Epidermal strips from the abaxial surface from leaves of 4‐ to 6‐week‐old plants were mounted in a home‐made chamber (Blatt, 1987; Wang, 2013). The epidermal peels were affixed with gentle contact to the glass bottom coated with an optically clear and pressure‐sensitive, silicone adhesive (no. 355 medical adhesive; Dow Corning). All impalements were carried out on a Zeiss IM35 inverted microscope (Zeiss, Germany) fitted with Nomarski Differential Interference Contrast optics.

K^+^ channel currents were recorded from guard cells bathed with 5 mM Ca^2+^‐2‐(N‐morpholino)ethanesulfonic acid (MES) (pH 6.1; 5 mM MES titrated to pH 6.1 with Ca(OH)_2_; [Ca^2+^] = 1 mM), plus an adequate volume of 1 M KCl to achieve final concentration between 0.1 and 30 mM. Continuous bathing solution flow was achieved using a gravity‐feed system and was removed by aspiration. Solution flow through the chamber was maintained at 5 mL/min (approx. 10 chamber volumes/min).

Tail protocols were performed on *G. gynandra* guard cells as follows. The impaled cell was first clamped to a high positive voltage until K^+^ channels were activated and stable. Then, the cells were stepped to voltages going from the same high positive voltage to a negative extreme. K^+^ channel currents were monitored as the curve relaxed to a new stable value (Blatt & Clint, 1989). The voltage in which the current relaxation direction reversed corresponded to *E*_K_. After extracting the curves using Henry's EP software, the *E*_K_ value was determined by quantifying directly from the relaxations at each potassium concentration.

The Nernst equation describes the electrical potential difference generated by the electrochemical gradients:

(4) $$E_{K}=\text{RT}/\text{zF}\times \;\log(x_{o}/x_{i}),$$

where *R* is the gas constant, *T* is the absolute temperature in degrees Kelvin, *z* is the charge on ion potassium, *F* is Faraday's constant, *x*_o_ is the potassium concentration inside the guard cell and *x*_i_ is the known potassium concentration outside.

To assess channel conductances and gating characteristics, recordings typically included a conditioning (holding) voltage at −200 mV (0.1 and 1 mM K^+^), −150 mV (3 mM K^+^) or −100 mV (10 and 30 mM K^+^) and eight to 10 steps to voltages between the holding voltage and +40 mV for K_out_, or −240 mV for K_in_. Steps were scaled in logarithmic mode to maximise the information yield with step lengths as described in figure legends. Surface areas of the impaled guard cells were calculated assuming a spheroid geometry from the orthogonal dimensions measured with an ocular micrometre (Blatt, 1987). Current–voltage analysis was carried out using Henry's EP software suite. Currents were corrected to the membrane surface area of guard cells and the time‐averaged steady‐state currents through K^+^ channels as a function of voltage (*I*–*V* curves) were calculated for each K^+^ concentration, [K^+^], after subtracting the instantaneous current (Blatt, 2004). Electrophysiological measurements of *Arabidopsis thaliana* guard cells followed similar process with modifications as described before (Chen et al., 2012).

Most impalements were successful when carried out on guard cells of partially or fully open stomata. G*. gynandra* plants 4‐ to 6‐week‐old usually presented cells that were stable for electrophysiology experiments. Younger leaves with three leaflets generally gave better measurements than older leaves with five leaflets. Stable recordings were generally obtained with mature guard cells with an average radius of 5.1 ± 0.1 and 27.8 ± 1.2 µm of length. By comparison, fully expanded leaves from 4 weeks‐old *A. thaliana* provided guard cells measuring 3.7 ± 0.2 in radius and 17.1 ± 1 µm in length, which yielded satisfactory impalements.

### 2.5 Statistical analysis

Data are presented as means ± SE of *n* observations, and *p* values were calculated using one‐way analysis of variance *p* < 0.05 followed by Tukey's multiple comparison test, *t* test (two‐tailed distribution and two‐sample equal variance), or as stated in the figure legend.

## 3 RESULTS

### 3.1 Gas exchange in the steady state and response to light and CO_2_

**Figure 1:**
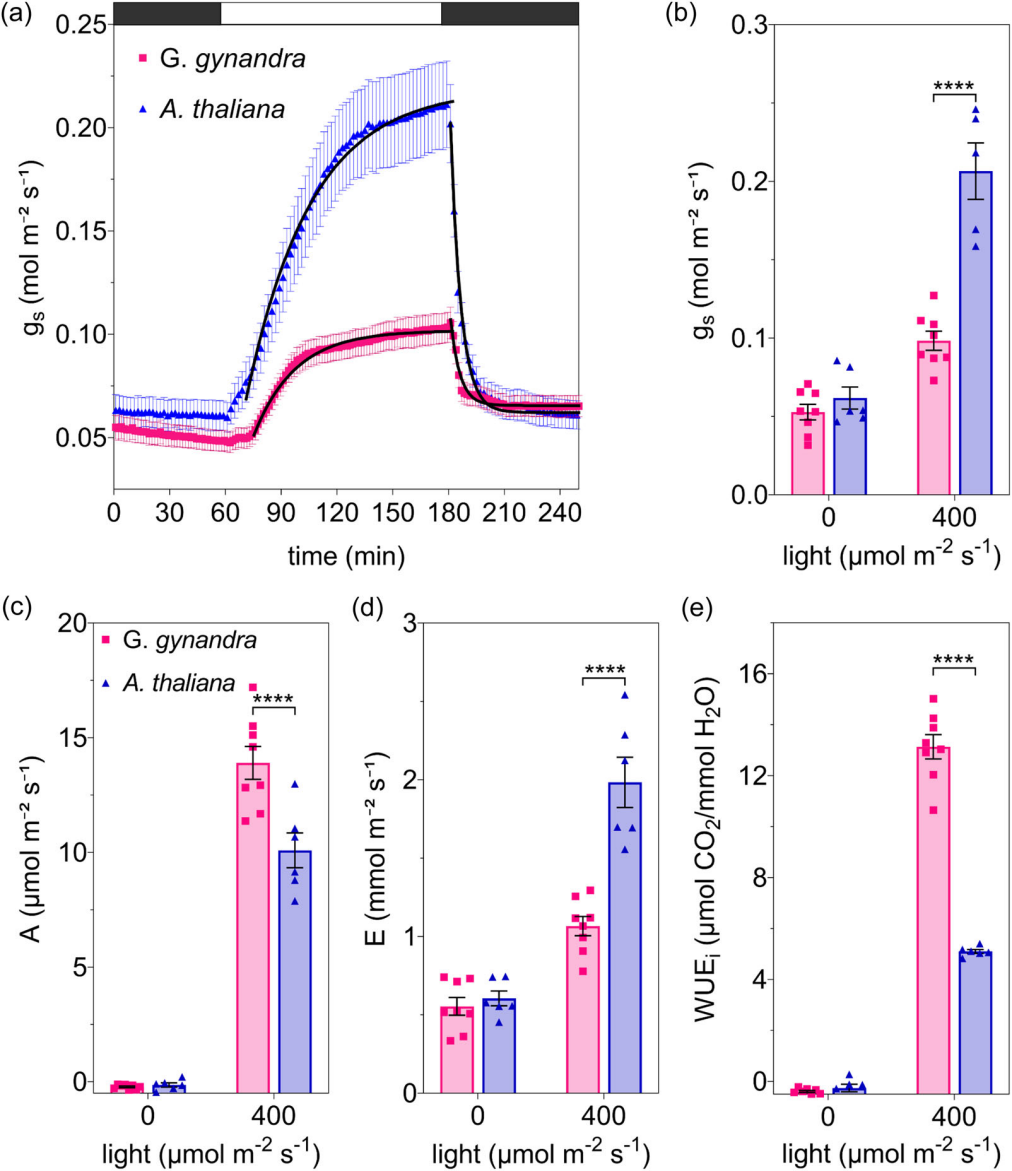
*Gynandropsis gynandra* has a low steady‐state stomatal conductance (*g*_s_) in the light. (a) Values for *g*_s_ of *Arabidopsis thaliana* (*n* = 6) and *G. gynandra* (*n* = 8) under different light intensity. The time scale in minutes is represented in the *x* axis, and *g*_s_ in mol m^−2^ s^−1^ in the *y* axis. Bar above indicates steps from dark to 400 µmol m^−2^ s^−1^. Plants were exposed to 400 µmol m^−2^ s^−1^ light for 2 h then transferred to dark for 2 h before the light step shown. For clarity, the time course omits every other data point. Solid lines are fittings to first‐order exponential functions (see Figure 3). (b–e) Panels are (b) *g*_s_, (c) Photosynthetic carbon assimilation (*A*), (d) transpiration (*E*) and (e) instantaneous water use efficiency (WUE_*i*_) calculated as *A*/*E*. Bars are for *G. gynandra* (pink squares; *n* = 8), and Arabidopsis (blue triangles; *n* = 6). Values are steady‐state means ± SE from *n* plants with individual measurements indicated by small symbols. Asterisks indicate significant differences (****p ≤ 0.0001) by post‐hoc unpaired *t* test.

**Figure 2:**
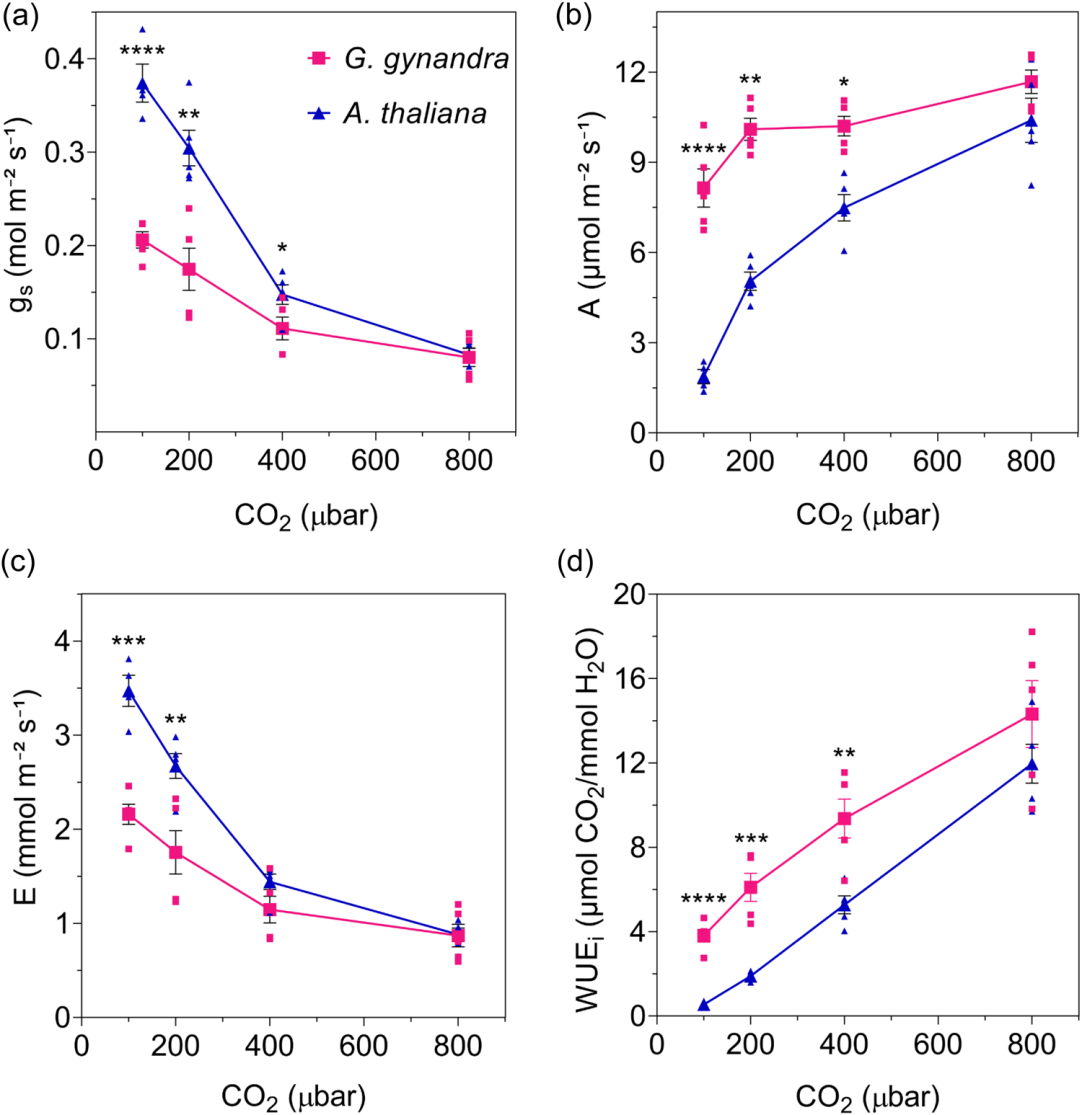
*Gynandropsis gynandra* has a low steady‐state stomata conductance (*g*_s_) under most CO_2_ treatments. Steady‐state means ± SE for *G. gynandra* (pink squares, *n* = 5) and *Arabidopsis thaliana* (blue triangles, *n* = 5) across CO_2_ partial pressures from 100 to 800 μbar for (a) *g*_s_, (b) photosynthetic carbon assimilation (*A*), (c) transpiration (E) and (d) instantaneous water use efficiency (WUE_*i*_) calculated as in Figure 1. Measurements were taken after 2 h stabilisation in each case. Small symbols are individual measurements. Asterisks indicate significant differences (*p ≤ 0.05, **p ≤ 0.01, ***p ≤ 0.001, ****p ≤ 0.0001) between plants at each CO_2_ partial pressure, with post‐hoc unpaired *t* test. [Color figure can be viewed at wileyonlinelibrary.com]

We measured gas exchange characteristics with fully expanded leaves of *G. gynanda* and Arabidopsis using steps in light and CO_2_ partial pressure to induce stomatal dynamics (Figures 1 and 2). Plants were routinely taken after 1 h of the start of the daylight period to allow for induction of Calvin cycle enzymes (Kirschbaum & Pearcy, 1988; Woodrow & Mott, 1992) and were adapted to dark for 2 h before the start of experiments. Values for *g*_s_ recorded from the C_4_ plant *G. gynandra* were generally near 50% of the values from Arabidopsis in the light (Figure 1, Table 1). The change in magnitude of the steady‐state *g*_s_ also differed, with values for Arabidopsis increasing in the light by more than threefold while in *G. gynandra g*_s_ rose by less than twofold (Figure 1b). We calculated the instantaneous WUE (WUE_*i*_) as the ratio between photosynthetic carbon assimilation (*A*) and transpiration rates (*E*). In the light, *G. gynandra* showed larger values for *A* and smaller values for *E* (Figure 1c,d), which translated to a threefold increase in WUE_*i*_ compared to Arabidopsis (Figure 1e). Qualitatively similar results were obtained under 200µmol m^−2^ s^−1^ of light (Supporting Information S1: Figure S1).

**Table 1:** Steady‐state gas exchange characteristics in the dark and light.

Light (µmol m^−2^ s^−1^)	*Gynandropsis gynandra* (*n* = 8)	*Arabidopsis thaliana* (*n* = 6)	*p* Value
*g*_s_ (mol m⁻² s⁻¹)			
0	0.05 ± 0.00	0.06 ± 0.01	ns
400	0.10 ± 0.01	0.21 ± 0.02	****
*A* (µmol m⁻² s⁻¹)			
0	−0.22 ± 0.03	−0.14 ± 0.09	ns
400	13.90 ± 0.72	10.09 ± 0.76	****
*E* (mmol m⁻² s⁻¹)			
0	0.55 ± 0.06	0.61 ± 0.05	ns
400	1.07 ± 0.06	1.98 ± 0.16	****
WUE (µmol CO_2_/mmol H_2_0)			
0	−0.40 ± 0.04	−0.26 ± 0.15	ns
400	13.13 ± 0.48	5.10 ± 0.08	****

*Note*: The stomatal conductance (*g*_s_), photosynthetic carbon assimilation (*A*), transpiration (*E*) and instantaneous water use efficiency (WUE = *A*/*E*). Data are means ± SE at 0, and 400 µmol m^−2^ s^−1^ light. Asterisks indicate significant differences (***p ≤ 0.0001); ns, not significant following post‐hoc unpaired *t* test.

Comparable results were obtained when changes in CO_2_ partial pressure were used to drive stomata opening and closing (Figure 2, Table 2). *G. gynandra* showed significantly lower *g*_s_ values with CO_2_ at and below 400 μbar (Figure 2a) and, again, showed higher values for *A* at all CO_2_ partial pressures below 800 μbar (Figure 2b). At 400 μbar, CO_2_ *G. gynandra* showed a twofold enhancement in WUE_*i*_ (Figure 2, Table 2).

**Table 2:** Steady‐state gas exchange characteristics with different CO_2_ partial pressures.

CO_2_ µbar	*Gynandropsis gynandra* (*n* = 5)	*Arabidopsis thaliana* (*n* = 5)	*p* Value
*g*_s_ (mol m⁻² s⁻¹)			
100	0.21 ± 0.01	0.37 ± 0.02	****
200	0.17 ± 0.02	0.31 ± 0.02	**
400	0.11 ± 0.01	0.15 ± 0.01	*
800	0.08 ± 0.01	0.08 ± 0.01	ns
*A* (µmol m⁻² s⁻¹)			
100	8.15 ± 0.64	1.88 ± 0.24	****
200	10.09 ± 0.37	5.05 ± 0.30	**
400	10.21 ± 0.33	7.49 ± 0.44	*
800	11.68 ± 0.39	10.40 ± 0.73	ns
*E* (mmol m⁻² s⁻¹)			
100	2.16 ± 0.11	3.47 ± 0.17	***
200	1.75 ± 0.23	2.67 ± 0.13	**
400	1.15 ± 0.14	1.44 ± 0.08	ns
800	0.87 ± 0.12	0.88 ± 0.07	ns
WUE (µmol CO_2_/mmol H_2_0)			
100	3.80 ± 0.31	4.98 ± 0.45	****
200	6.10 ± 0.67	17.46 ± 0.93	***
400	9.36 ± 0.93	51.66 ± 4.20	**
800	14.32 ± 1.59	127.90 ± 9.16	ns

*Note*: Stomatal conductance (*g*_s_), photosynthetic carbon assimilation (*A*), transpiration (*E*) and instantaneous water use efficiency (WUE = *A*/*E*). Data are means ± SE (*n* = 5) at of 100, 200, 400 and 800 µbar CO_2_. Asterisks indicate significant differences between species (*p ≤ 0.05, **p ≤ 0.01, ***p ≤ 0.001, ****p ≤ 0.0001); ns, not significant following post‐hoc unpaired *t* test.

Stomata distribution and anatomy is an important characteristic that influences carbon fixation and WUE. We compared stomata density and size from abaxial peels from 4‐week‐old plants of *G. gynandra*, and Arabidopsis (Supporting Information S1: Figure S2). Overall, *G. gynandra* presented bigger leaves with a marginal 20% lower stomatal density compared to the C_3_ model Arabidopsis (Supporting Information S1: Figure S2a,b). However, stomata of *G. gynandra* were substantially larger, about 20% greater length and twofold greater in width when compared with Arabidopsis (Supporting Information S1: Figure S2c), indicating that lower steady‐state *g*_s_ values of *G. gynandra* were not a consequence of these static properties of the stomata.

**Figure 3:**
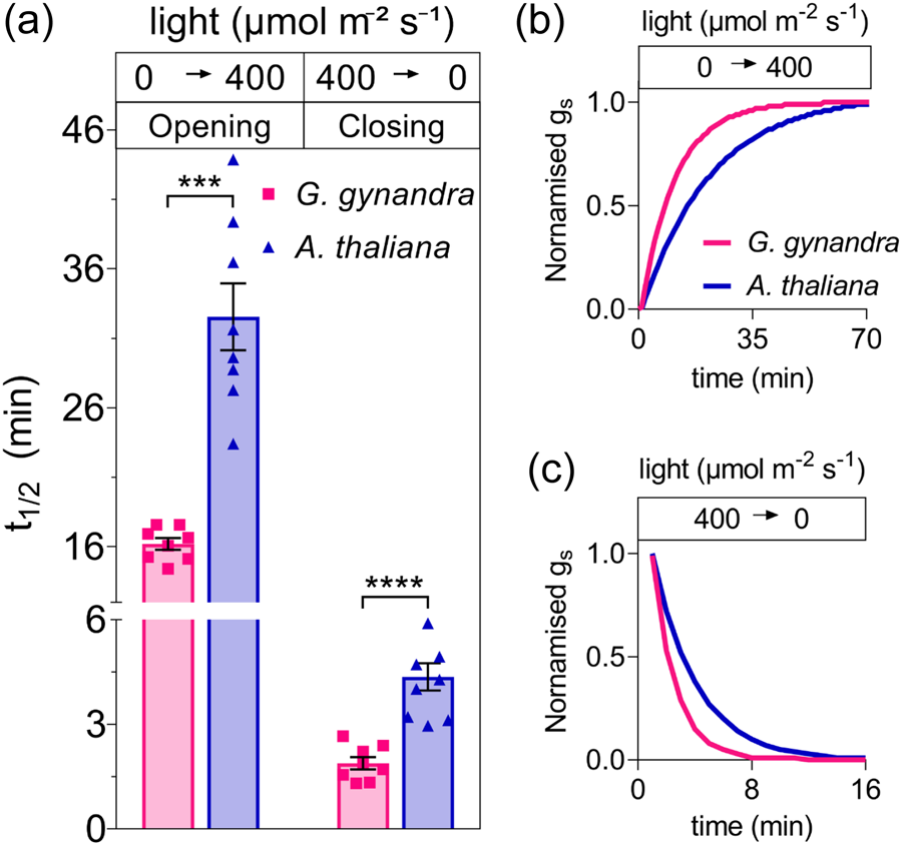
Stomata of *Gynandropsis gynandra* are faster than Arabidopsis on light transitions. (a) Halftimes (*t*_1/2_) for *G. gynandra* (red bars; *n* = 8), and *Arabidopsis thaliana* (blue bars; *n* = 6) determined by fitting data as in Figure 1 to first‐order exponential functions (see Section 2). Data are means ± SE with symbols indicating individual measurements. Asterisks indicate significant differences (***p ≤ 0.001, ****p ≤ 0.0001) with post‐hoc unpaired *t* test. (b, c) Panels show the fitted *g*_s_ curves after normalisation to scale the changes in Figure 1 to a common dynamic range. [Color figure can be viewed at wileyonlinelibrary.com]

Because our measurements showed differences in *g*_s_ dynamics when varying external light and CO_2_ partial pressure (Figures 1 and 2), we examined whether *G. gynandra* stomata were faster to close and open compared with Arabidopsis. To quantify the kinetic differences while changing environmental conditions, we fitted the *g*_s_ relaxations on opening and closing to a first‐order exponential function (see Section 2). The analysis showed that the stomata of the C_4_ plant were roughly twofold faster both in opening and closing with transitions between light and dark (Figure 3). Closing halftimes were 1.9 ± 0.2 min for *G. gynandra* and 4.4 ± 0.4 min for Arabidopsis. Conversely, when varying CO_2_ partial pressure (Supporting Information S1: Figure S3), the primary difference was seen with opening, *G. gynandra* being some threefold faster when CO_2_ was stepped to lower partial pressures.

### 3.2 Characteristics of the outward‐rectifying K^+^ channels

**Figure 4:**
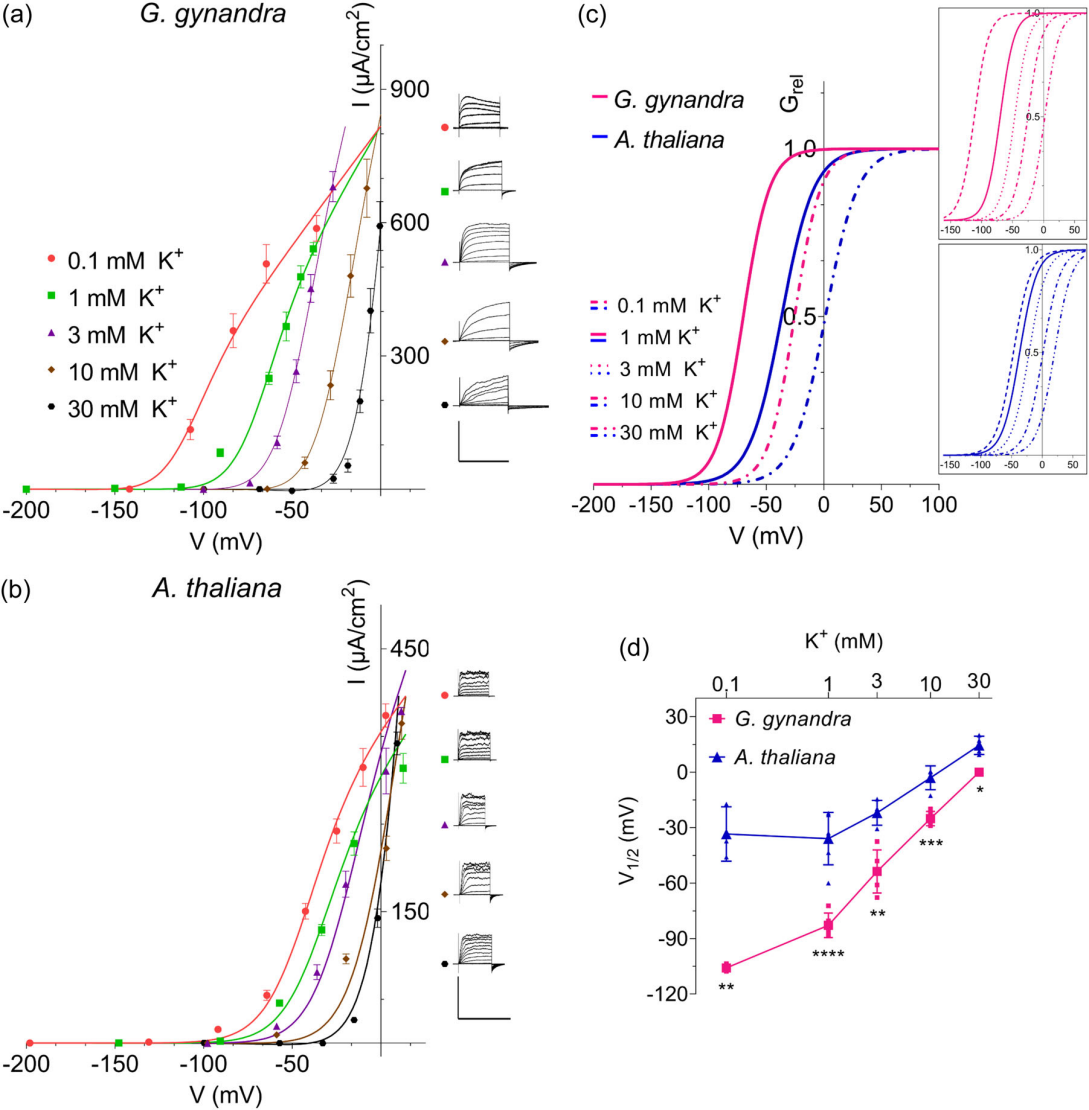
Outward‐rectifying K^+^ channels of *Gynandropsis gynandra* guard cells gate at voltages negative of those for the channels of Arabidopsis. (a) Steady‐state current–voltage (*I*–*V*) from *G. gynandra* (*n* = 5) guard cells superfused with 5 mM Ca^2+^‐MES, pH 6.1, with 0.1,1, 3, 10 and 30 mM KCl. Currents were recorded under voltage clamp from conditioning voltages of −200 mV (0.1 and 1 mM KCl), −150 mV (3 mM) and −100mV (10, and 30 mM KCl) to test voltage steps from the conditioning voltage to +40 mV. Tailing voltage steps were the same as the conditioning voltages. Steady‐state *I*–*V* curves were calculated from currents recorded at the end of the test steps after subtracting the instantaneous background at the beginning of each test step. Curves were fitted jointly to a Boltzmann function (Equation 5) by nonlinear least‐squares minimisation using a Marquardt–Levenberg algorithm. Fittings yielded a common *δ* of 1.97 ± 0.1 and the *V*_1/2,_ and *G*_max_ values listed in Table 3. Current traces from representative guard cells. Scale bar: 10 s (horizontal), 800μA cm^−2^ (vertical). (b) Steady‐state *I*–*V* curves from Arabidopsis (*n* = 5) guard cells superfused with the same solutions and clamp protocols as in (a). Fittings by nonlinear least‐squares minimisation (above) yielded *δ* of 1.78 ± 0.1 and *V*_1/2,_ and *G*_max_ values in Table 3. Current traces from representative guard cells. Scale bar: 10 s (horizontal), 500 μA cm^−2^ (vertical). (c) Relative conductance–voltage (Grel–*V*) curves calculated from fitted mean steady‐state currents of *G. gynandra* (pink lines) and Arabidopsis (blue lines) of (a) and (b) at 1 and 10 mM KCl using Equation (6). Grel–*V* curves across the full range of KCl concentrations. (d) Conductance midpoint voltage (*V*_1/2_) as a function of external K^+^ concentration. Values were taken from the fittings in (a) and (b) Data are means ± SE with asterisks indicating significant differences (*p ≤ 0.05, **p ≤ 0.01, ***p ≤ 0.001, ****p ≤ 0.0001) with post‐hoc unpaired *t* test. [Color figure can be viewed at wileyonlinelibrary.com]

Two‐electrode voltage clamp measurements were carried out using double‐barrelled microelectrodes filled with 200 mM K^+^‐acetate, pH 7.5 (Blatt, 1987). Stable recordings were generally obtained with mature guard cells of *G. gynandra* and the results were calculated on the basis of cell surface area (Supporting Information S1: Figure S4). Clamping the guard cell membrane of *G. gynandra* and Arabidopsis, from a conditioning voltage negative of *E*_K_ to more positive values, yielded time‐ and voltage‐dependent currents characteristic of outward‐rectifying K^+^ channels described previously (Blatt & Gradmann, 1997; Horaruang et al., 2022). As is typical of these channels, increasing external K^+^ shifted the steady‐state current–voltage (*I*–*V*) curves to more positive voltages and slowed current activation at any one voltage (Figure 4a,b).

To assess the selectivity and [K^+^] dependence of the channels, tail currents were analysed from *G. gynandra* guard cells by stepping to more negative voltages after activating the current. As expected of a K^+^‐selective channel, increasing K^+^ outside displaced the current reversal voltage to the right (Supporting Information S1: Figure S5). *G. gynandra* guard cells yielded reversal voltages of −153 ± 8, −118 ± 4, −97 ± 3 and −59 ± 6 mV, when superfused with 0.1, 1, 3 and 10 mM KCl, respectively (Supporting Information S1: Table S1). A plot of these data showed that, from 1 mM K^+^ and above, the reversal potentials followed a Nernstian slope, displaying a +58 mV shift for each 10‐fold increment in [K^+^] that indicated a high selectivity for K^+^ (Supporting Information S1: Figure S5b). The near‐Nernstian response also accords with the free‐running membrane voltage and its dependence on extracellular K^+^, confirming that the cation dominates the overall membrane conductance.

To understand the kinetics of gating of the outward‐rectifying channels, we calculated the channel activation halftimes (*t*_1/2_) as functions of membrane voltage and external [K^+^]. Values for *t*_1/2_ were calculated directly from the current traces and represent the times at which half of the maximum steady‐state current was achieved (Blatt, 1988). Current activation in *G. gynandra* and in Arabidopsis guard cells responded to the membrane voltage and external [K^+^] much as previously described for outward‐rectifying K^+^ channels in *Vicia*, tobacco and Arabidopsis guard cells (Armstrong et al., 1995; Blatt, 1988; Blatt & Gradmann, 1997; Eisenach et al., 2014; Grabov et al., 1997). Increasing the membrane voltage led to a decrease in the *t*_1/2_ for channel activation in both species, while increasing [K^+^] in the bath solution shifted the curves to the right, to more positive values. (Supporting Information S1: Figure S6).

The Boltzmann function:

(5) $$I=G_{\max}(V-E_{K})/(1+e^{\delta F(V‐V_{1/2})/\text{RT}}),$$

is commonly used to compare gating properties from different channels (Blatt & Gradmann, 1997; Jezek et al., 2021; Wang et al., 2012). Here *I* is the current, *G*_max_ is the maximum ensemble conductance, *V* is the voltage, *E*_K_ is the equilibrium voltage for K^+^ across the membrane, *V*_1/2_ is the voltage at which the ensemble conductance *G* equals 0.5. *G*_max_, *δ* is the voltage sensitivity coefficient for gating, and *F*, *R* and *T* have their usual meanings. We fitted *I*–*V* curves jointly to Equation (5) to obtain the curves plotted with the experimental data (Figure 4a,b). The fittings showed that the *G*_max_ for the *G. gynandra* channels was roughly twofold greater than for Arabidopsis (Supporting Information S1: Figure S7), and they also showed a distinct negative shift to the *I*–*V* curves.

To resolve the shift in the *I*–*V* curves, the relative conductance–voltage (*G*_rel_–*V*) relations are plotted in Figure 4c. This formulation allows an understanding of channel gating with membrane voltage that is independent of the ion gradient across the membrane. The *G*_rel_–*V* curves were determined from the above parameter values as:

(6) $$G_{\text{rel}}=1/(1+e^{\delta F(V‐V_{1/2})/\text{RT}}).$$

This analysis clearly shows a substantial negative shift in the *G. gynandra* conductance when compared with Arabidopsis.

Fittings to the Boltzmann function (Equation 5) also provide a reference for the K^+^ sensitivity of the channel gate in the [K^+^] dependence of *V*_1/2_. We found a mean shift in the *V*_1/2_ of 56 ± 8 mV/K^+^ decade for *G. gynandra*, and a shift of 34 ± 8 mV/K^+^ decade for Arabidopsis (Figure 4d). Across all [K^+^] analysed, *V*_1/2_ values for *G. gynandra* were significantly more negative than the values for Arabidopsis (Figure 4d, Table 3), the difference being most pronounced at the lower range of external K^+^ concentrations. For instance, the *G. gynandra V*_1/2_ was around 72 mV more negative at 0.1 mM K^+^, and 47 mV more negative at 1 mM K^+^ than the *V*_1/2_ values for Arabidopsis. At 30 mM K^+^ this difference decreased to 14 mV. These characteristics are a strong indicator for a voltage dependence in the affinity for K^+^ action on gating.

**Table 3:** Characteristics of outward‐rectifying K^+^ channels.

	K^+^ (mM)	*Gynandropsis gynandra* (*n* = 5)	*Arabidopsis thaliana* (*n* = 5)	*p* Value
*V*_1/2_ (mV)	0.1	−106 ± 1	−33 ± 8	**
	1	−83 ± 3	−36 ± 6	****
	3	−54 ± 5	−22 ± 3	**
	10	−25 ± 2	−3 ± 3	***
	30	0 ± 0	14 ± 2	*
*G*_max_ (mS cm^−2^)	0.1	7 ± 2	3 ± 1	**
	1	9 ± 2	4 ± 0.5	**
	3	12 ± 1	7 ± 0.4	**
	10	16 ± 2	13 ± 1	ns
	30	25 ± 6	23 ± 2	ns

*Note*: Maximum conductance (*G*_max_), and conductance midpoint voltage (*V*_1/2_) from guard cells superfused with 5 mM Ca^2+^‐MES, pH 6.1, with 0.1, 1, 3, 10 and 30 mM of KCl. Asterisks indicate significant differences between species (*p ≤ 0.05, **p ≤ 0.01, ***p ≤ 0.00).

**Figure 5:**
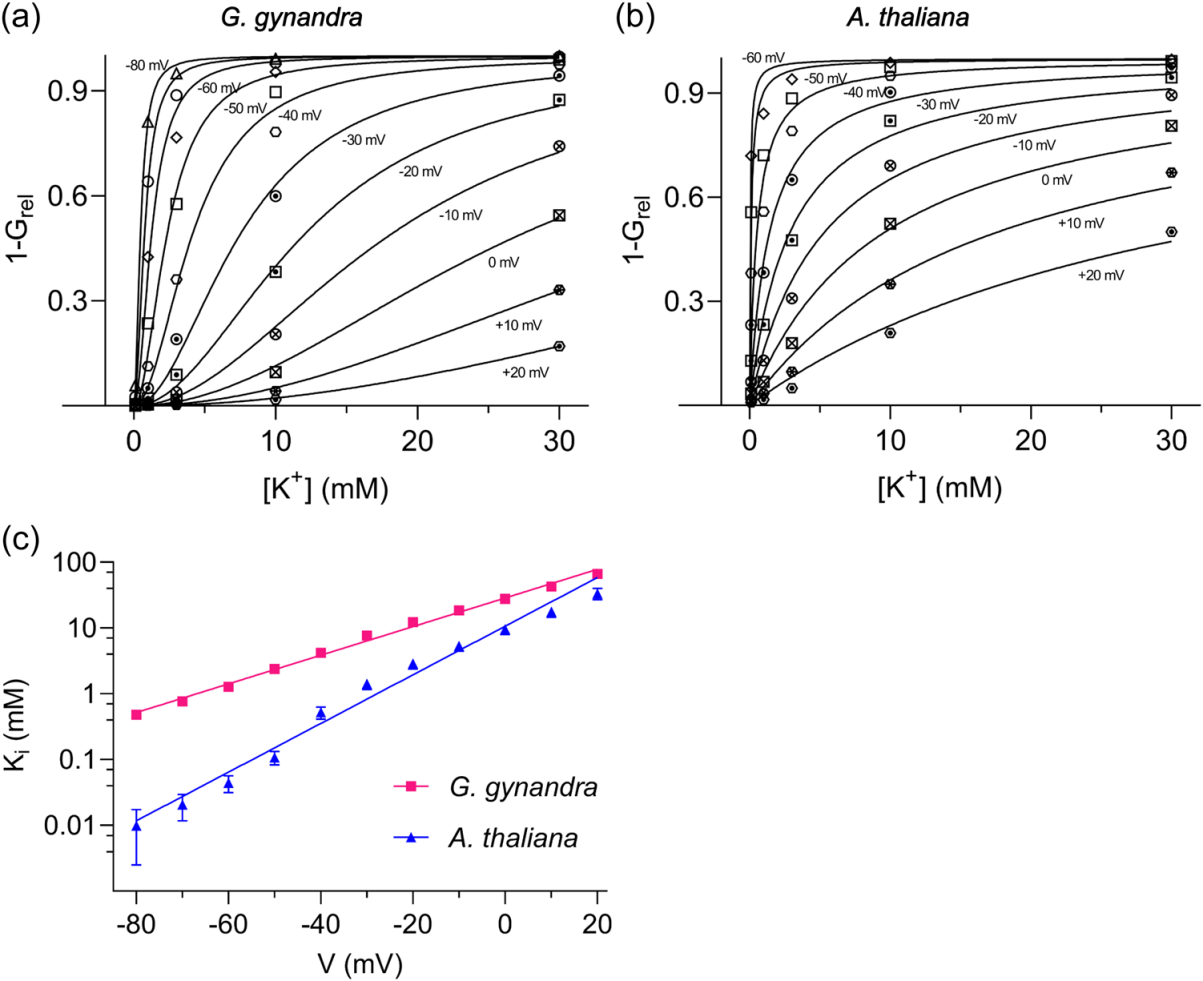
The apparent K_*i*_ for K^+^ inhibition of gating is voltage‐dependent and differs between species. (a, b) Relative conductance data from Figure 1 transformed as the conductance complement, 1 − *G*_rel_, and plotted as a function of K^+^ concentration at voltages from −80 to +20 mV. Data are for (a) *Gynandropsis gynandra* and (b) Arabidopsis. Solid lines are joint nonlinear least‐squares fitting to the Hill Equation (8) with the Hill coefficient *n* held in common between data sets and only K_*i*_, allowed to vary with voltage. Best fittings were obtained with a value of 2 for *G. gynandra* and 1 for *Arabidopsis thaliana*. (c) Values of K*_i_* from (a) and (b) plotted as a function of voltage. Linear regression analysis (solid lines) indicates an *e*‐fold decrease in apparent affinity for K^+^ per 56.1 mV for *G. gynandra* and 25.4 mV for Arabidopsis. [Color figure can be viewed at wileyonlinelibrary.com]

To compare the apparent affinities for gating inhibition by external K^+^, K_*i*_, we transformed the data of Figure 4c after normalising to *G*_max_ to derive its complement, 1–*G*_rel_. Plots of the conductance complement as a function of external [K^+^] yielded families of binding curves (Figure 5a,b) that were well‐fitted jointly to the Hill Equation (Blatt & Gradmann, 1997):

(7) $$1-G_{\text{rel}}=\left({\left[K^{+}\right]}^{n}\right)/\left(K_{i}^{n}+{\left[K^{+}\right]}^{n}\right),$$

where K_*i*_ is the apparent constant for inhibition, and *n* is the Hill coefficient. Best fittings (Figure 5c, solid lines) indicated an *e*‐fold rise in apparent K_*i*_ for K^+^ with (−)56.1 mV for *G. gynandra* and (−)25.4 mV for Arabidopsis. In other words, the apparent voltage sensitivity of inhibition for the channels of *G. gynandra* is roughly half of that for Arabidopsis. Over the physiological range of voltages, say at 1 mM [K^+^] and −60 mV, channels of *G. gynandra* showed a 3.6‐fold lower affinity for inhibition by K^+^ ions than in Arabidopsis.

### 3.3 Characteristics of the inward‐rectifying K^+^ channels

Finally, we asked whether *G. gynandra* might present inward‐rectifying K^+^ channels that could contribute to K^+^ uptake and whether these channels were comparable to Arabidopsis or exhibited differences in their gating properties. To address these questions, we recorded inward‐directed currents when clamping the membrane to voltages negative of the K^+^ equilibrium voltage, much as described above. Inward‐rectifying K^+^ channels that have been described in the guard cells of *Vicia*, tobacco and Arabidopsis are typically activated at voltages negative of −150 mV which, in millimolar K^+^, provides a driving force for K^+^ flux directed into the cell (Jezek & Blatt, 2017). While the *G*_max_ of these channels increases directly with K^+^ outside, the wild‐type current generally has shown no shift in *V*_1/2_ (Blatt, 1992; Hertel et al., 2005; Thiel et al., 1992).

Clamping *G. gynandra* and Arabidopsis guard cells from conditioning voltages of −100 or −120 mV to more negative values yielded the characteristic sigmoidal inward‐directed K_in_ currents (Supporting Information S1: Figure S8). As before, currents were corrected for membrane surface area. We found that increasing K^+^ outside led to a scalar increase in current, but we observed no obvious shift in the steady‐state *I*–*V* curves. Again, we fitted the currents jointly to the Boltzmann function (Equation 5) to extract the gating characteristics. The analysis (Supporting Information S1: Table 2) showed no significant difference in *V*_1/2_, either as a function of K^+^ outside or in comparison to the current characteristics for Arabidopsis. The only difference was in the absolute magnitude of *G*_max_, with values in 10 mM K^+^ roughly 80% greater in *G. gynandra* when compared with Arabidopsis.

## 4 DISCUSSION

Although the C_4_ pathway is present in only 3% of flowering species, it contributes approximately 25% of primary biomass production on land (Sedelnikova et al., 2018; Still et al., 2003). C_4_ plants cope better with high temperature and light, and with limited water supply. These are factors at the forefront of concerns around climate change (Yadav & Mishra, 2020). In addressing these concerns, much attention has focused on photosynthetic pathways and carbon fixation and on the associated macroscopic features of stomata. Thus, it is generally recognised that stomata of C_4_ plants function over a dynamic range in CO_2_ that differs substantially from that of C_3_ plants (Aubry et al., 2016; Israel et al., 2022; Osborne & Sack, 2012; Zhao et al., 2022). Yet, our understanding of what properties of the guard cells are altered to adjust stomatal mechanics in C_4_ plants remains fragmentary at best.

Stomatal mechanics are determined, first and foremost, by the characteristics of pumps and channels that facilitate ion flux across the guard cell plasma membrane. Prominent among these transporters are two classes of channels that enable K^+^ flux in and out of the guard cell. Studies across a range of species, albeit primarily C_3_ plants, have long demonstrated major roles for K^+^ channels that are biased, or rectify, for K^+^ uptake and activate when H^+^‐ATPases hyperpolarise the membrane, and those that rectify for K^+^ loss when the membrane depolarises, driven by inactivation of the H^+^‐ATPases and activation of anion channels and Cl^−^ efflux (Jezek & Blatt, 2017). Even so, there is almost no quantitative detail pertaining to the characteristics of the K^+^ channels of C_4_ species, and what little information is available in the literature pertains almost exclusively to the dumbell‐shaped guard cells of *Zea mays* (Bauer et al., 2000; Fairley‐Grenot & Assmann, 1993; Gao et al., 2017; Natura & Dahse, 1998; Philippar et al., 1999, 2003; Terry et al., 1992).

Recent work with Arabidopsis (Blatt & Alvim, 2022; Horaruang et al., 2022; Jezek et al., 2021) has highlighted the importance of K^+^ channels and their regulation in the speediness of stomatal responses to light and to CO_2_. Against this background, we asked whether the unique properties of stomata in plants of a C_4_ species might be linked to the characteristics of one or more of the K^+^ channels. Here we show that the enhanced kinetics of stomata in the C_4_ plant *G. gynandra* correlate with the gating characteristics of the outward‐rectifying K^+^ channels present in the guard cells. By comparison with its close relative Arabidopsis, the channels in *G. gynandra* present substantially greater activities and, most important, altered dependencies on membrane voltage and a reduced sensitivity to external K^+^ for the outward rectifying channels. These characteristics, like those of the N‐terminally modified GORK channels described for Arabidopsis, offer a natural explanation for the rapid kinetics observed in the C_4_ plants.

### 4.1 *G. gynandra* maintains lower and faster stomata conductance responses

As expected, we observed lower rates of steady‐state transpiration, *g*_s_ and higher WUE for *G. gynandra* when compared with Arabidopsis (Figures 1 and 2, Tables 1 and 2). These results reinforce much published work showing that many C_4_ species achieve carbon assimilation with higher WUE and lower *g*_s_ than related C_3_ lineages (Israel et al., 2022; Osborne & Sack, 2012; Zhao et al., 2022). Unexpected, however, were the faster stomata movements in response light and CO_2_ steps (Figure 3, Supporting Information S1: Figures S1 and S3) and the mismatch when comparing stomatal densities and size. In general, it has been commonly held that stomatal *g*_s_ is positively correlated with stomatal density and negatively correlated to stomatal size (Franks & Beerling, 2009; Harrison et al., 2020). Usually, reducing the size of the guard cells has the effect of increasing the ratio of membrane surface area to guard cell volume (Franks & Farquhar, 2007), which might be expected to promote faster stomata opening and closing (Lawson & Blatt, 2014). Although larger in size, stomata of *G. gynandra* were substantially faster and presented lower *g*_s_ values under varying light and CO_2_ (Figures 1, 2, 3; Supporting Information S1: Figures S1–S3). These results are consistent with previously reports demonstrating that stomata of C_4_ species often open and close faster than C_3_ species (Huxman & Monson, 2003; Israel et al., 2022; McAusland et al., 2016; Ozeki et al., 2022).

It is of interest that stomata from the C_4_ species of *Flaveria*, like *G. gynandra*, also show a small dynamic range in *g*_s_ (Huxman & Monson, 2003). Similarly, Israel et al. (2022) confirmed that C_4_ grasses operate with a much lower *g*_s_ relative to the maximal stomatal conductance, than do C_3_ grasses. The reduced dynamic range is likely to arise because C_4_ photosynthesis operates at a much reduced CO_2_ within the leaf airspace (*C*_i_), but this behaviour clearly indicates altered patterns of stomatal control (Israel et al., 2022; Vogan & Sage, 2012). Previously research also observed that *A* and *g*_s_ were significantly influenced by CO_2_, light and photosynthetic type (Huxman & Monson, 2003; Israel et al., 2022), which are consistent with results presented here (Tables 1 and 2).

These parallels notwithstanding, the enhanced stomatal kinetics must also contribute to the water use and assimilation efficiencies of *G. gynandra*. Broadly speaking, improved coordination between *A* and *g*_s_ avoids unnecessary transpiration rather than limiting CO_2_ uptake for photosynthesis (Israel et al., 2022; McAusland et al., 2016; Nobel, 2020; Ozeki et al., 2022). Rapid stomatal opening favours CO_2_ assimilation, and accelerated closure will save water, improving WUE when light becomes limiting for photosynthesis (Horaruang et al., 2022; Lawson & Blatt, 2014; Ozeki et al., 2022; Papanatsiou et al., 2019). In short, the relationships between stomatal dynamics, *g*_s_, *A* and *E* are not adequately explained by stomatal anatomy and density.

### 4.2 Enhanced gating of outward‐rectifying K^+^ channels in *G. gynandra*

Electrophysiological recordings from *G. gynandra* yielded characteristics in general agreement with those from the guard cells other plants (Blatt & Gradmann, 1997; Jezek & Blatt, 2017; Thiel et al., 1992). Analysis of the *G. gynandra* K^+^ channels indicated a high selectivity for K^+^ as the permeant ion, and both the inward‐ and outward‐rectifying currents exhibited the characteristic voltage‐dependencies described for the functional homologues in several C_3_ species (Blatt, 1988; Blatt & Gradmann, 1997; Jezek & Blatt, 2017; Thiel et al., 1992). Likewise, the K^+^ independence in gating of the inward‐rectifying current and the K^+^ dependence in gating of the outward‐rectifying current proved the qualitative equivalents of the channel currents in these other species (Ache et al., 2000; Blatt, 1988, 1992; Blatt & Gradmann, 1997; Eisenach et al., 2014; Jezek & Blatt, 2017; Thiel et al., 1992).

A singular feature between these guard cell K^+^ channels, however, was a negative shift in K^+^ dependence for gating of the outward‐rectifier in *G. gynandra* (Figures 4 and 5; Table 3). Against the backdrop of *G. gynandra* stomatal kinetics, the −20 to −60 mV shift in *V*_1/2_ marks a very substantial reduction in the free energy for gating that, at voltages between −60 and −80 mV, manifests as 36‐ to 50‐fold decreases in the K*_i_* for gating inhibition by the cation. For purposes of comparison, the difference in gating free energy (ΔΔ*G*) between *G. gynandra* and Arabidopsis is defined as:

(8) $$\Delta \Delta G=-F(\delta_{\text{At}}\times V_{1/2,\text{At}}-\delta_{\text{Gg}}\times V_{1/2,\text{Gg}}),$$

where *V*_1/2_ and *δ* values were derived from joint fittings of the *I*–*V* curves to the Boltzmann function (Figure 4, Table 3). The comparison shows that the energy required for gating of the *G. gynandra* channel was reduced by −1.07 Kcal/mol relative to Arabidopsis at −60 mV in 10 mM K^+^. These findings are important because they compare favourably with the −40 mV shift in GORK gating that Horaruang et al. (2022) identified as necessary and sufficient to accelerate, by roughly 1.5‐ to 1.8‐fold, both stomatal closing and opening in Arabidopsis.

How can enhancing the gating of an outward‐rectifying K^+^ channel accelerate both closing and opening? Modelling with the systems platform OnGuard3 (Horaruang et al., 2022; Jezek et al., 2021; Wang, Hills, et al., 2014; Wang, Noguchi, et al., 2014) and experiments in vivo (Horaruang et al., 2022) have shown that reducing the affinity for gating inhibition by K^+^ (Figure 5), and thereby shifting voltage dependence of gating even −25 to −30 mV, introduces a new K^+^ conductance to the membrane. Most important, this conductance appears within the normal physiological range, both positive and negative of the K^+^ equilibrium voltage, *E*_K_. Positive of *E*_K_ the effect is to promote cation efflux for stomatal closure; negative of *E*_K_ the effect is to facilitate K^+^ uptake for stomatal opening. So, the added conductance enhances stomatal kinetics, reducing the time‐averaged values for *E* while promoting *A* under fluctuating light.

It is worth noting that the differences in gating of the outward‐rectifying channels of *G. gynandra* and Arabidopsis almost certainly dominate over any differences in current amplitudes (Table 3; Supporting Information S1: Figures S7 and S8). Wang, Hills, et al. (2014) and Wang, Noguchi, et al. (2014) reported that overexpressing, even by factors of three‐ to fivefold, two plasma membrane K^+^ channels in guard cells of Arabidopsis had no effect on *g*_s_ or WUE. Modelling also leads to the same conclusion: any impacts of simple scalar increases in channel current are largely suppressed in simulation by counteracting adjustments in free‐running membrane voltage that affect channel activities (Wang, Hills, et al., 2014; Wang, Noguchi, et al., 2014).

### 4.3 Implications for C_4_ engineering

The operational parallels between the outward‐rectifying K^+^ channels of *G. gynandra* and the gating mutants of GORK, identified by Horaruang et al. (2022) must also beg questions about their molecular basis in *G. gynandra*, whether these characteristics are common among a wider range of C_4_ species, and how these characteristics might be harnessed, both to enhance photosynthesis in existing C_4_ crops and to support efforts towards engineering C_4_ photosynthesis within C_3_ crop species. These are challenges that come with recognising global climate change (NOAA, 2022) and the certain increase in demands for food (Blatt et al., 2022; Gupta et al., 2020).

Horaruang et al. (2022) identified as critical to K^+^ gating inhibition sets of alternating positive and negative charges within an intrinsically disordered region of the GORK N‐terminus. This domain and charge alternations are conserved among a number of putative, outward‐rectifying K^+^ channels in the guard cells of C_3_ species, including rice and wheat. How this domain affects channel gating has yet to be fully resolved, but the knowledge is sure to help guide efforts to engineer the channels. Equally, it remains now to be seen whether similar intrinsically disordered regions are present in the K^+^ channels of *G. gynandra* and other C_4_ species and, if so, how these differ to confer an elevated K_*i*_ for gating inhibition by K^+^ to shift the voltage dependence of the channels.

Finally, it is worth considering the characteristics of stomata in developing novel crops with C_4_ photosynthesis. To date, much effort has gone into strategies for introducing C_4_ traits in plants that carry out C_3_ photosynthesis, for example, establishing a synthetic Kranz anatomy (Ermakova et al., 2020), biochemical ‘pumps’ to concentrate CO_2_ around RuBisCO in modified bundle sheath cells (Ermakova et al., 2021; Lin et al., 2020), and directing phosphoenolpyruvate carboxylase expression and that of related C_4_ enzymes to promote yield under water restriction (Giuliani et al., 2019; Gu et al., 2013). Very little consideration has gone to the likely need to match carbon assimilation with stomatal responsiveness. Yet, it is clear that adapting stomatal functionality is sure to be important in securing the maximum gain from such engineering efforts (Ermakova et al., 2020; Lawson & Blatt, 2014; McAusland et al., 2016; Ozeki et al., 2022; Schlüter & Weber, 2020).

In summary, our work describing the characteristics of the *G. gynandra* outward‐rectifying K^+^ channels shows that relatively subtle changes in the biophysical properties of a key transporter may have very significant impacts that are directly relevant to C_4_ engineering. Our findings highlighted distinct gating properties of the C_4_ plant *G. gynandra* that differ from those of Arabidopsis and other C_3_ species studied to date. These differences correlate with the accelerated stomata kinetics of *G. gynandra*. Without doubt, there will be other characteristics of C_4_ guard cell transport, biophysical as well as regulatory, that will surface and need to be taken into account together to enhance stomatal kinetics and benefit fully in future engineering efforts.

## Supporting information

Supporting information.

## Data Availability

The data that support the findings of this study are available from the corresponding author upon reasonable request.
