# Supplementary material for: Speedy stomata of a C4 plant correlate with enhanced K+ channel gating
Source: Plant Cell Environ. 2023 Nov 27;47(3):817–31. doi: 10.1111/pce.14775 (PMC10953386; doi:10.1111/pce.14775)
Supplement: Supplementary file 1 — Supporting information. [file PCE-47-817-s001.pdf]

# Speedy stomata of a $C_4$ plant correlate with enhanced $K^+$ channel gating

Fernanda A.L. Silva-Alvim[1]*; Jonas Chaves Alvim[1], Andy Harvey[2]; Michael R. Blatt[1]

[1]Laboratory of Plant Physiology and Biophysics, Bower Building, and
[2]Physics & Astronomy, Kelvin Building, University of Glasgow, Glasgow, G12 8QQ, UK
*Contact for editorial communication: fernanda.alvim@glasgow.ac.uk

Short title: $K^+$ channels of *Gynandropsis*

## Supplementary Tables and Figures

**Supplementary Table S1. Outward-rectifying $K^+$ channel reversal voltages for *G. gynandra* and Arabidopsis.**
Reversal voltages measured from guard cells of *G. gynandra* acquired using clamp protocol as described previously in Supplementary Figure 5, and published data for Arabidopsis.

| $E_K$ | $K^+$ (mM) | V (mV) | Reference |
|---|---|---|---|
| ***G. gynandra*** | 0.1 | -153 ± 8 | This paper |
| | 1 | -118 ± 4 | |
| | 3 | -97 ± 3 | |
| | 10 | -59 ± 9 | |
| ***A. thaliana*** | 1 | -113 ± 12 | (Colombo and Cerana, 1991; Maathuis and Sanders, 1993; Ivashikina et al., 2001) |
| | 10 | -60, -68 | |

**Supplementary Table S2. Gating of Inward-rectifying $K^+$ channels in G. gynandra and Arabidopsis are independent of external $K^+$.**
Comparison of the midpoint voltages ($V_{1/2}$) and maximum conductance ($G_{max}$) values for the data of Figure S8. Data are means ±SE. Asterisks indicate significant differences (*** $p \leq 0.001$); ns, no significant difference after post-hoc unpaired t test between plants between plants.

| | $K^+$ (mM) | *G. gynandra* | *A. thaliana* | *p value* |
|---|---|---|---|---|
| **$V_{1/2}$ (mV)** | 1 | -197 ± 16 | -201 ± 6 | *ns* |
| | 3 | -220 ± 3 | -200 ± 4 | *ns* |
| | 10 | -218 ± 5 | -211 ± 8 | *ns* |
| **$G_{max}$ (mS $cm^{-2}$)** | 1 | 0.5 ± 0.0 | 0.6 ± 0.2 | *ns* |
| | 3 | 1.3 ± 0.1 | 1.1 ± 0.3 | *ns* |
| | 10 | 5.5 ± 0.2 | 3.3 ± 0.9 | *** |

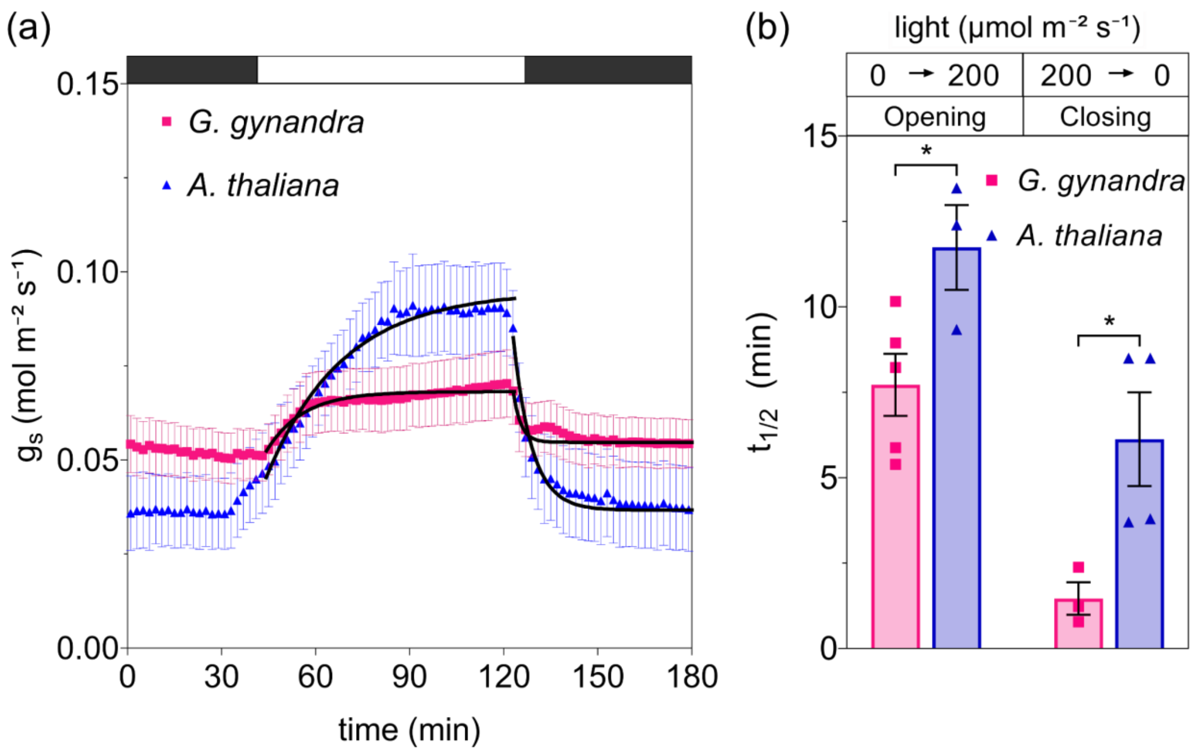


**Supplementary Figure S1: *G. gynandra* stomata respond rapidly with light steps between 0 to 200 µmol $m^{-2}s^{-1}$.**

(a) Time course of stomatal conductance ($g_S$) for Arabidopsis (n=4) and *G. gynandra* (n=5) with light steps from dark to 200 µmol $m^{-2}s^{-1}$. Plants were pretreated as described in Figure 1. Bar (above) indicates times of light transitions. For clarity only every other data point is plotted here. Solid curves are the results of non-linear least-squares fittings to first-order exponential functions. (b) Mean halftimes ($t_{1/2}$) ±SE for stomatal conductance relaxations in (A) derived from the fittings for *G. gynandra* (red bars; n=5), and Arabidopsis (blue bars; n=4). Symbols are individual measurements. Asterisks indicate significant differences (* $p \leq 0.05$) following post-hoc unpaired *t test*.

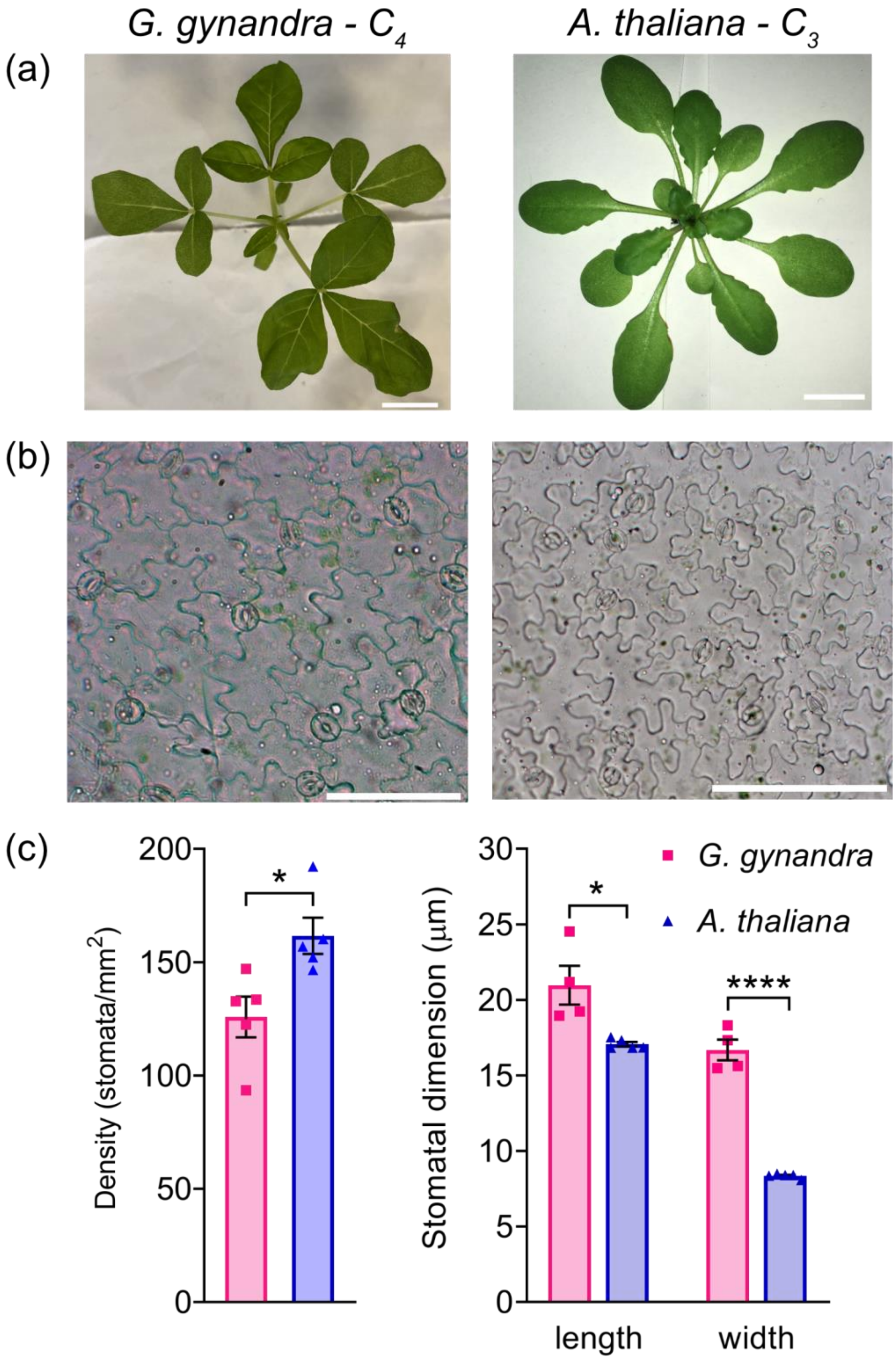


**Supplementary Figure S2. Growth and stomatal characteristics for *G. gynandra* and Arabidopsis.**
(a) Representative plants of *G. gynandra* (left) and Arabidopsis (right) after 4-weeks of growth. Scale bars: 1cm. (b) Abaxial epidermal peels showing stomata for *G. gynandra* (left) and Arabidopsis (right). Scale bars: 100 µm. (c) Means ±SE for stomatal density (left) of *G. gynandra* (pink squares; n = 4) and Arabidopsis (blue triangles; n=5). Means ±SE for stomatal length, and width for both plants. Symbols are individual measurements. Asterisks indicate significant differences (*, $p < 0.05$; ****, $p<0.0001$) between plants, after post-hoc unpaired *t test*.

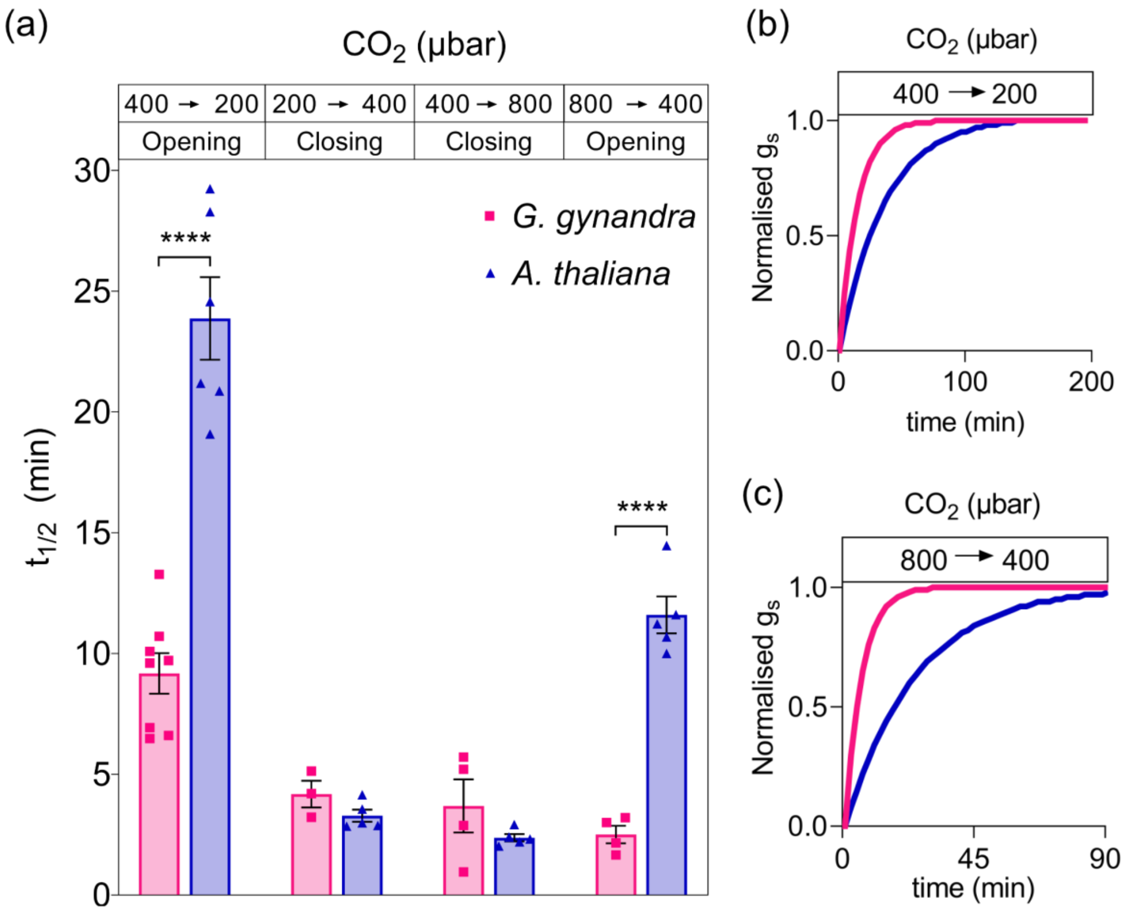


**Supplementary Figure S3: *G. gynandra* shows rapid stomatal opening with $CO_2$ steps.**

(a) Mean halftimes ($t_{1/2}$) ±SE for *G. gynandra* (red bars; n=5) and Arabidopsis (blue bars; n=5) for stomatal response to $CO_2$ steps between 400, 200 and 800 µbar. Data are from non-linear least-squares fitting to data from Figure 2, to exponential functions. Symbols are individual measurements. Asterisks indicate significant differences (****p ≤ 0.0001) between plants at each $CO_2$ partial pressure, after post-hoc unpaired t test. (b,c) Panels show the corresponding fitted relaxations after normalizing.

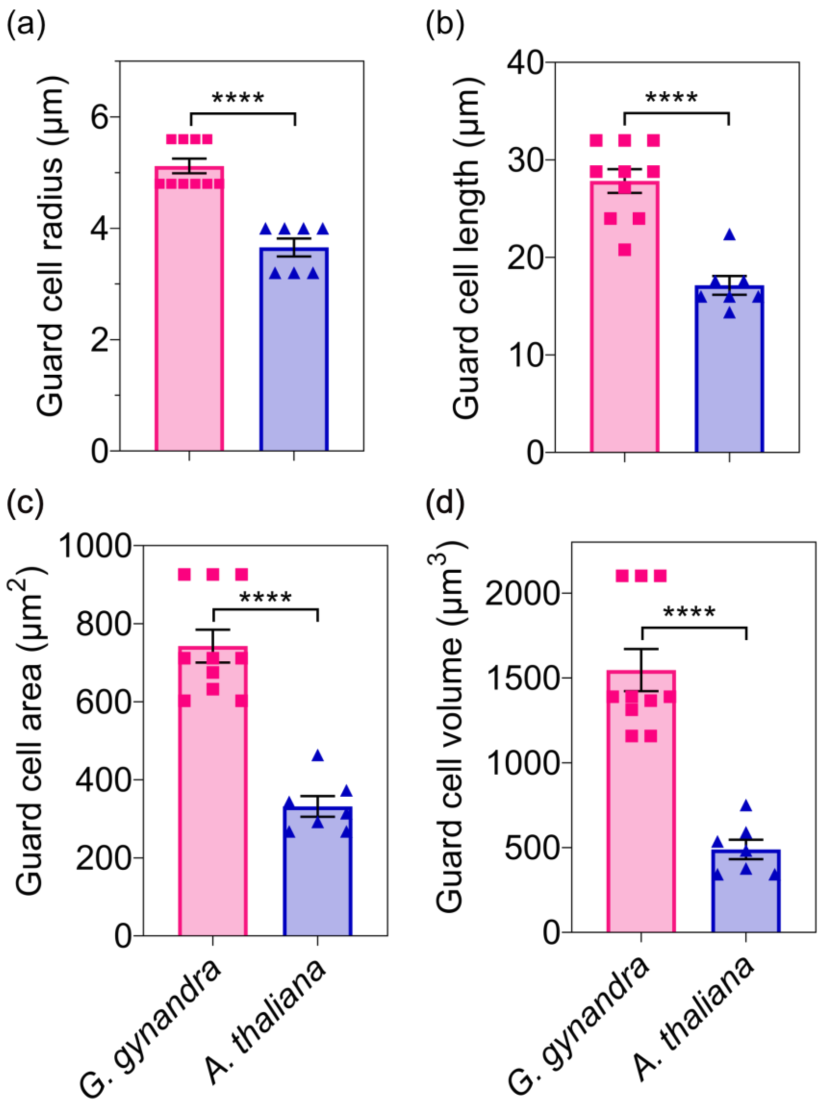


**Supplementary Figure S4*. *Stomatal characteristics for impaled guard cells.** (a,b) Means ±SE for *G. gynandra* (n = 10) and Arabidopsis (n = 7) guard cell (a) radius and (b) length measured directly from impaled guard cells. Symbols are individual measurements. (c,d) Means ±SE for (c) guard cell surface area and (d) volume calculated using Henry's EP suite. Asterisks indicate significant differences ($p < 0.05$) between plants, after post-hoc unpaired *t test*. Symbols are individual measurements.

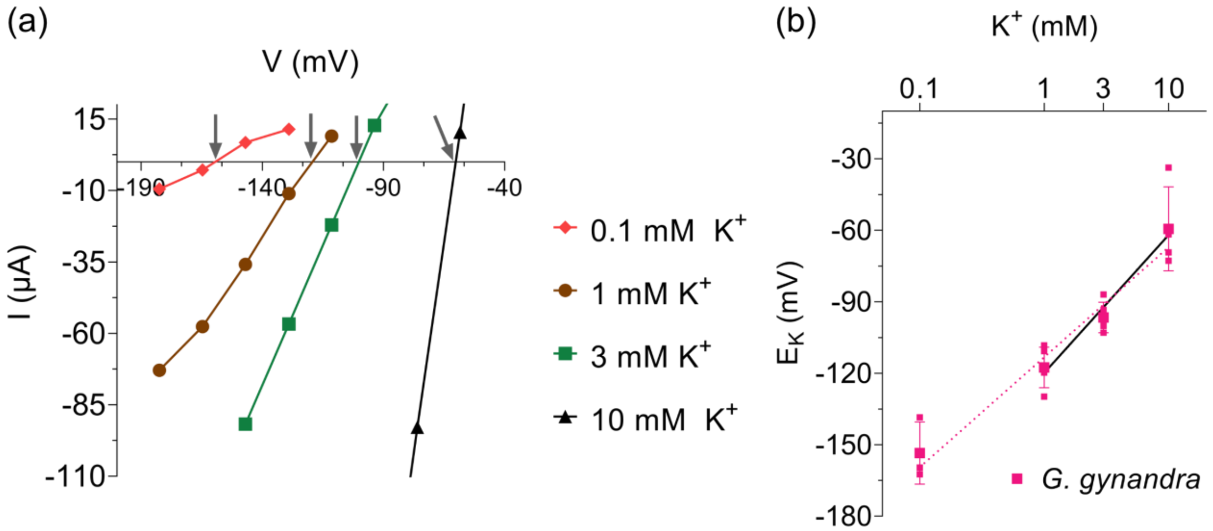


**Supplementary Figure S5. *G. gynandra* outward-rectifier $K^+$ channels are highly selective for $K^+$.**

Tail current analysis for current reversal voltages from guard cells superfused with 5 mM $Ca^{2+}$-MES, pH 6.1, with different concentrations 0.1, 1, 3 or 10 mM KCl. Cells were clamped at +30 mV to activate the outward-rectifying $K^+$ channels and then stepped to voltages from +30 and -200 mV. (a) Representative tail current amplitudes from one guard cell plotted as a function of clamp voltage. Reversal voltages are indicated by the grey arrows where the current-voltage curve crosses the voltage axis. For clarity only points close to x-axis are shown. (b) Mean reversal voltages ±SE (n ≧ 6) plotted as a function of external $K^+$ concentration. The dashed line through the points gives a slope of 46 mV/[$K^+$] decade, and the black line gives near-Nernstian 58 mV/$K^+$ decade for data points of 1 mM $K^+$ and above. Both lines are non-linear least-squares fittings to a log-linear function. Small symbols are individual measurements with larger symbols for the means ±SE.

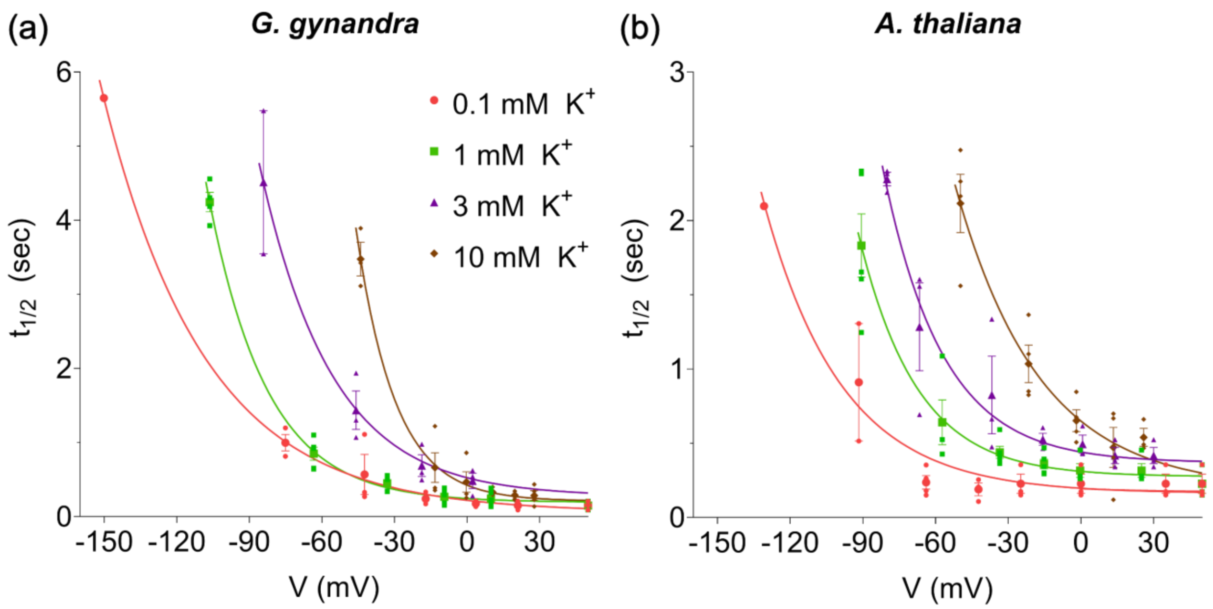


**Supplementary Figure S6. Activation of the outward-rectifying $K^+$ channels of *G. gynandra* guard cells is potassium- and voltage-dependent.**
Comparison of activation halftimes ($t_{1/2}$) as a function of membrane voltage in guard cells of (a) *G. gynandra* (n = 5) and (b) Arabidopsis superfused with 5 mM $Ca^{2+}$-MES, pH 6.1, with 0.1, 1, 3, and 10 mM KCl. Halftimes ($t_{1/2}$) ±SE are shown as the larger symbols, with small symbols indicating individual measurements. Solid lines are non-linear least-squares fittings to a single exponential decay function. Note the curves are displaced to the right along the voltage axis with increasing $K^+$.

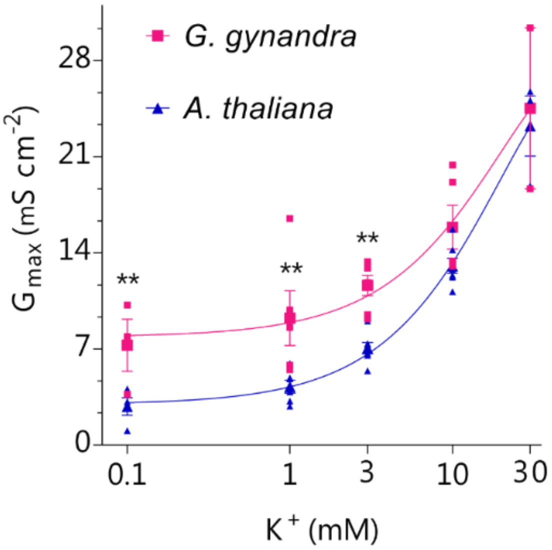


**Supplementary Figure S7. *G. gynandra* guard cells showed elevated $G_{max}$ values by comparison with Arabidopsis.**

Maximum conductance ±SE ($G_{max}$) from *G. gynandra* (red, n = 5) and Arabidopsis (blue, n = 5) guard cells. Data are from Figure 4 with small symbols for individual measurements and larger symbols for means ±SE. Asterisks indicate significant differences ($p < 0.05$) between plants, after post-hoc unpaired *t test*. The solid lines are fitting to a first-order exponential function and are included as a visual guide only.

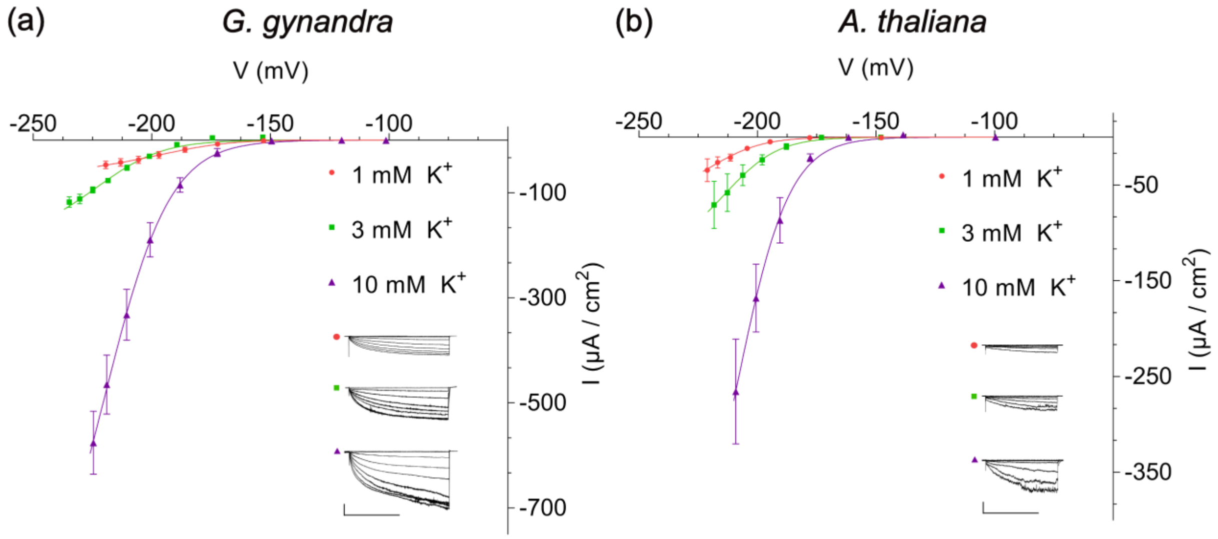


**Supplementary Figure S8. Gating of the inward-rectifying $K^+$ channels of *G. gynandra* and Arabidopsis are not altered by $K^+$ outside.**
Steady-state current-voltage (I-V) from (a) *G. gynandra* (n = 6) and (b) Arabidopsis (n = 6) guard cells superfused with 5 mM $Ca^{2+}$-MES, pH 6.1, with 1, 3, and 10 mM KCl. Currents recorded by clamping cells with conditioning voltages at -120 mV (1 and 3 mM KCl) or -100mV (10 mM KCl), followed by steps negative to values between conditioning voltage and -240 mV before returning to the conditioning voltage. Steady-state I-V curves were calculated from currents at the end of the test steps after subtracting the instantaneous current at the start of each test step. Curves are joint, non-linear least-squares fittings to the Boltzmann function (Eqn [1]). Fittings yielded parameters for *G.gynandra*: δ, -1.9±0.1; $G_{max}$, 0.15±0.1 (0.1), 0.43±0.1 (3), and 1.73±0.8 mS/cm$^2$ (10 mM $K^+$); for Arabidopsis: δ, -2.2±0.1 $G_{max}$, 0.45±0.1 (1), 0.86±0.3 (3), and 3.24±0.9 mS/cm$^2$ (10 mM $K^+$). Values for $V_{1/2}$ are listed in Supplementary Table 2. Insets: Current traces from representative guard cells. Scale bar: 5 sec (horizontal), 100 µA/cm$^2$ (vertical).
